# A virtual patient authoring tool for transcatheter aortic valve replacement

**DOI:** 10.1007/s11548-024-03293-x

**Published:** 2024-12-19

**Authors:** Seyedsina Razavizadeh, Markus Kofler, Matthias Kunz, Joerg Kempfert, Ruediger Braun-Dullaeus, Janine Weidling, Bernhard Preim, Christian Hansen

**Affiliations:** 1 Faculty of Computer Science, University of Magdeburg, Magdeburg, Germany; 2 Department of Cardiothoracic and Vascular Surgery, Charité Berlin, Berlin, Germany; 3 Clinic for Cardiology and Angiology, University of Magdeburg, Magdeburg, Germany; 4 Siemens Healthcare GmbH, Erlangen, Germany

**Keywords:** Virtual reality, Medical training, Structural heart disease, Per-vertex animation

## Abstract

**Purpose:**

Computer-based medical training scenarios, derived from patient’s records, often lack variability, modifiability, and availability. Furthermore, generating image datasets and creating scenarios is resource-intensive. Therefore, patient authoring tools for rapid dataset-independent creation of virtual patients (VPs) is a pressing need.

**Methods:**

An authoring tool and a virtual catheterization laboratory environment were developed. The tool allows customised VP generation through a real-time morphable heart model and Euroscore parameters. The generated VP can be examined inside the vCathLab using a fluoroscopy and monitoring device, both on desktop and immersive virtual reality. Seven board-certified experts evaluated the proposed method from three aspects, i.e. System Usability Scale, qualitative feedback, and its performance in VR.

**Results:**

All participants agreed that this method could provide the necessary information and is anatomically correct within an educational context. Its modifiability, variability, and simplicity were well recognised. The prototype achieved excellent usability score and considerable performance results.

**Conclusion:**

We present a highly variable VP authoring tool that enhances variability in medical training scenarios. Although this work does not aim to explore didactic aspects, the potential of using this approach in an educational context has been confirmed in our study. Accordingly, these aspects can benefit from a thorough investigation in the future. In addition, our tool can be improved to provide more realistic parameter ranges for procedure-specific cases.

**Supplementary Information:**

The online version contains supplementary material available at 10.1007/s11548-024-03293-x.

## Introduction

In their latest curriculum revision, the European Association of Percutaneous Cardiovascular Interventions (EAPCI) recommend including simulation-based training in the curriculum of all education institutes [1]. However, access to patient data and creating virtual patients (VPs) is a slow, challenging, and expensive process [2]. Due to their reality-based nature, their modifiability is limited [2, 3]. Furthermore, these simulations are computationally expensive and bound to specific hardware, which impacts their availability. This leads to limited training scenarios, hence reducing the long-term engagement with the system.

To fulfil the training goals of interventional cardiology procedures, e.g. transcatheter aortic valve replacement (TAVR), simulation methods have been introduced, both commercially and academically, that provide VP models [4, 5]. These approaches aim to prepare the users for procedural workflows, focusing on risk management and decision-making [6]. Research on simulation-based training has yielded promising results across different fields, such as cardiovascular intervention [7], TAVR procedures [8], Coronary angiography [9], and cardiology care [10, 11].

Authoring tools allow experts to define training scenarios using predefined elements, thus increasing variability [12, 13] With the advancement of machine learning, some of these tools have achieved higher levels of realism. Furlan et al. introduced a VP simulator that uses natural language processing to help trainees improve their diagnostic reasoning through real-life interactions and appropriate feedback from the VP [14].

Mixed reality technologies can provide a higher degree of availability, modifiability, safety, and cost reduction compared to their conventional alternatives [1, 15]. Mao et al. observed that implementing virtual reality (VR) for medical education has considerably enhanced the exposed group performance [15]. Subsequently, Perez-Gutierrez et al. introduced a VR-based training scenario for acute myocardial infarction [16]. Li et al. proposed a percutaneous cardiovascular interventions simulation system that generates a simulated scenario based on patient datasets [17]. However, the research in this field started only recently, probably due to the high computational demands of medical simulation scenarios in a VR environment.

Hence, we investigate an alternative approach to address the limitations of conventional VP development methods by using a parameterisable patient model. This authoring tool explores the possibility of a rapid and straightforward way to generate a wide variety of patient cases with low effort and costs while maintaining context-relevant anatomical correctness. Additionally, we explore whether our method can be implemented in a computationally low-cost and resource-friendly fashion.

## Materials and methods

**Fig. 1:**
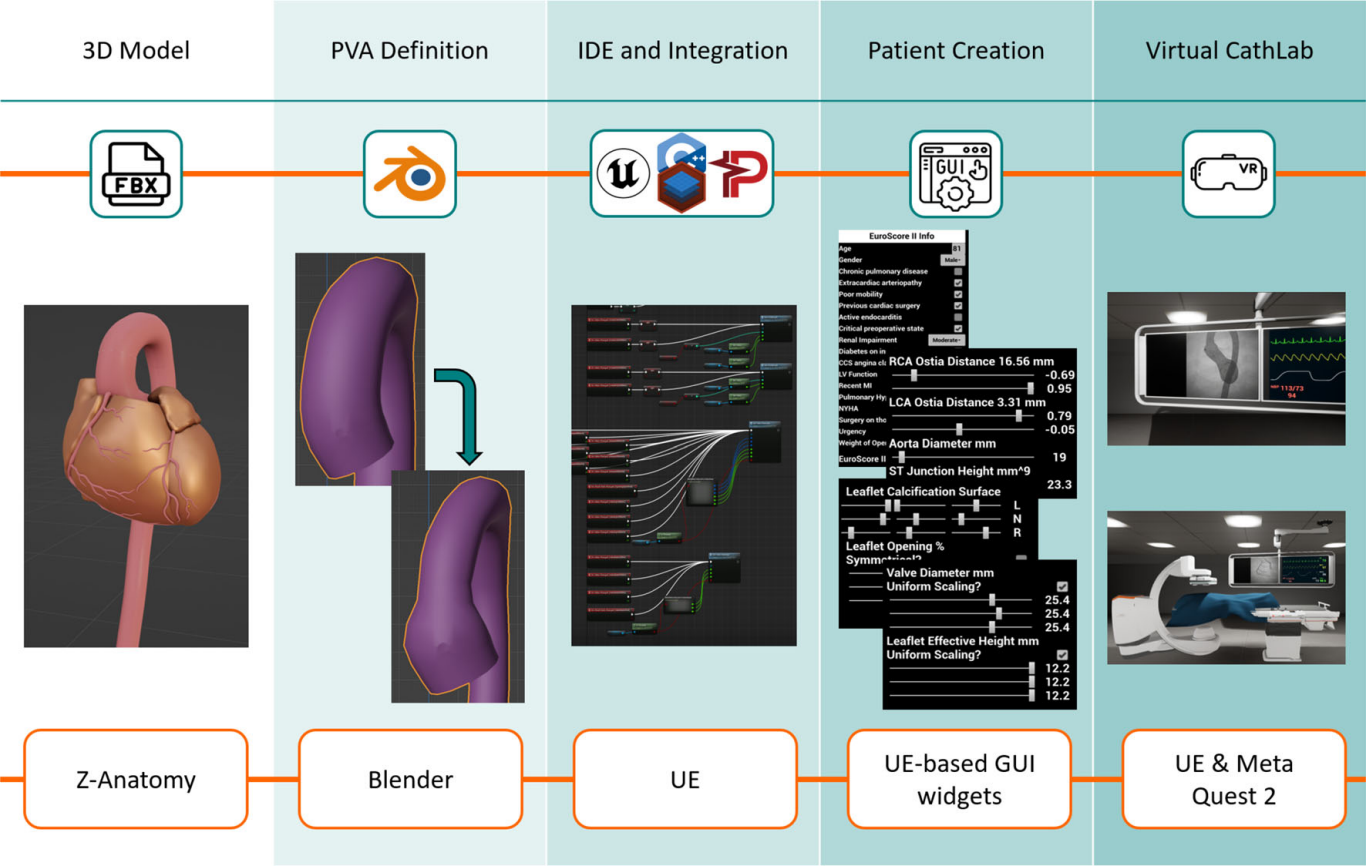
Overview of our virtual patient authoring tool

We propose an authoring tool pipeline consisting of a morphable animated 3D model, algorithms to dynamically limit the value ranges, an automatic patient generator, an interface with a physiology simulator, and a virtual catheterisation laboratory (vCathLab) environment to display the model in VR (Fig. 1).

### Experimental setup

We used a desktop PC with NVIDIA GeForce RTX 3080, Intel Core i9-10th gen, and 32GB of RAM. Blender was used (Blender Foundation, USA) for our 3D modelling and content creation. Unreal Engine 5.0.3 (Epic Games, USA) was selected for this work. A Meta Quest 2 (Meta, USA) device was connected via Quest Link and interfaced via SteamVR (Valve Corporation, USA) for the VR development. The physiology of the patient is simulated using Pulse Physiology Engine (PPE) (Kitware, USA).

### Identification of anatomical features

Understanding the role of the anatomical structures in disease and treatment is one of the leading training goals in TAVR education [18]. Hence, providing reliable information during the training is critical. To achieve a comparable result with patient-data-dependent models, we identified the major anatomical features impacting a TAVR procedure, from the treatment selection to operation [18]. Using medical literature, the mean and range of these variables were determined (see Table 1).

**Table 1:** List of anatomical features, their measurements, and relative PVA group information

Anatomical feature	Literature	Range based on literature	Implemented range	Limitation dependency	PVA group
Aorta diameter	Tadros et al. [19]	20 – 37*mm*	18 – 32*mm*	–	Aorta Diameter
RCA distance	Piazza et al. [20]	$17.2\pm 3.3mm$ from the base of the right leaflet	7.7 – 16.7*mm* from the VA	ST Height, Valve Diameter	ST Height, Valve Diameter, RCA orifice U/D/L/R, RCA U/D/L/R
LCA distance		$14.4\pm 2.9mm$ from the base of the left leaflet	0.1 – 5.9*mm* from the VA	ST Height, Valve Diameter	ST Height, Valve Diameter, LCA orifice U/D/L/R, LCA U/D/L/R
ST height	PCR Online [21]	$20.3\pm 3.3mm^9$ from leaflets base	17.3 – $23.3mm^9$	–	ST Height
Leaflet calcification surface	Gollman-Tepekoylu et al. [22]	Calcification pattern on the leaflet surface	Full situational control with three sliders per leaflet	–	Achieved via Physic-based Rendering Material
Leaflets opening		Entirely open to completely shut, represented as a percentage	0 – 100% separately for each leaflet	Leaflet Calcification Pattern	Left Coronary Leaflet, Non-Coronary Leaflet, Right Coronary Leaflet
Valve diameter	Blanke et al. [23]	$20.3\pm 2.1mm$ (17.7 – 29.3)	18.1 – 28.2*mm*	–	Coronary Leaflet, Non-Coronary Leaflet, Right Coronary Leaflet, ST Diameter, VA Diameter, RCA and LCA Position
Valve basal ring diameter		The diameter of the left ventricle outflow tract is based on the size of the valve and the virtual ring	6.8 – 24.4*mm*	Valve Diameter, Leaflet Effective Height, VA Height	–
Leaflet effective height	Jelenc M et al. [24]	$8.5\pm 1.4mm$ (4.8 – 14.0)	17.5 – 12.2*mm*	Valve Diameter	Left Coronary Leaflet, Non-Coronary Leaflet, Right Coronary Leaflet
VA height	–	This value is the distance from the valve hinges to the virtual basal ring distance and is calculated based on the largest effective height of the valve’s leaflets	7.6 – 18.9*mm*	Valve Diameter, Leaflet Effective Height, Valve Basal Ring Diameter	VA Height

U/D/L/R up/down/left/right

### Anatomical dataset

We used the Z-anatomy anatomical model library (Z-Anatomy, Gauthier Kervyn) as the basis for our 3D heart model. This library provides a variety of 3D models of healthy organs and systems based on previously acquired medical images. We included the aorta (up to abdominal bifurcation), coronary arteries, heart muscle, left and right atrium, aortic valve, and left ventricle. These meshes were further modified in Blender to fulfil our needs, e.g. polygon reduction, improving the valves polygon count, refining the ventriculoarterial junction (VA), isolating the left ventricle from the heart muscle, creating the sinotubular junction (SA) bulging, and assigning separate materials to the aorta, each valve leaflet (inner and outer surfaces), left ventricle, heart muscle, left and right coronary arteries (LCA and RCA). Further modifications are discussed below (see Sect Morphing of Anatomical Features).

#### Morphing of anatomical features

**Fig. 2:**
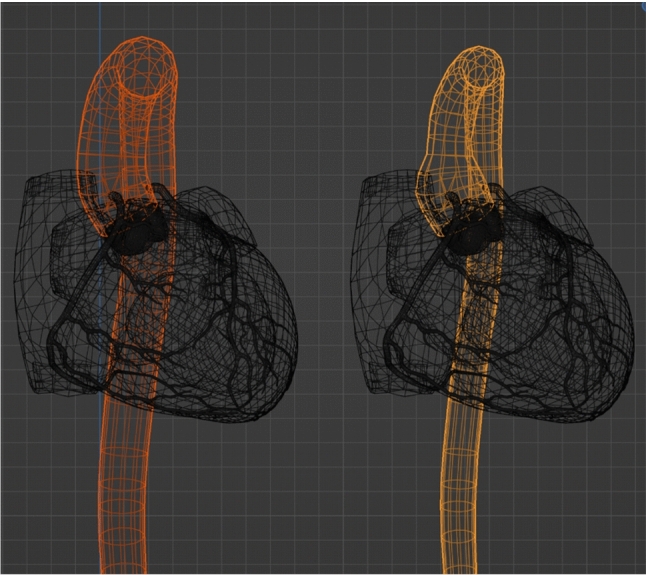
A PVA example for the aorta in orange colour, prepared in Blender. Aorta PVA interpolation value, $left = 0$ $right=0.7$ normalised between 0 and 1

Per-vertex animation (PVA) is an established method for vertex-level manipulation of a 3D model, for instance in facial animations. In this method, the location of the vertices are controlled by interpolating between two predefined locations using an interpolation value [25]. We implemented PVA for our heart model based on the values in Table 1 (see Fig. 2). Using the in-app measuring tool, we measured each feature (e.g. diameter, height, circumference) while applying our vertex modification to achieve realistic results. These values do not cover the whole reported ranges due to limitations of the heart model. While this excludes extreme cases, it still covers a wide variety of anatomies. To improve PVA malleability, some anatomical features were broken down into multiple PVA groups, namely in the ostia of the arteries, which consist of a separate shifting group for up, down, left, and right. Furthermore, adjusting the vertices in smaller groups, in combination with manual implementation of PVA values for the extreme cases, ensures that minimum distortions and self-intersections appear in the model after changing PVA values.

PVA offers computationally cheap and highly detailed animation sequences by saving different values on a timeline. We used this method to create animation sequences for the beating of the heart muscle, coronary arteries, left ventricle, and aorta. While these sequences are synced visually, their values are accessible and can be adjusted separately. This offers the opportunity for modifiable heart animations based on the simulated patient of “PPE”, such as rapid pacing.

### Calculation of surgical risk

Surgical risk scores are one criterion for selecting the proper treatment for each patient. A widely used scoring system is Euroscore II, which indicates the mortality rate in heart surgeries for adult patients [26]. Considering this score, the heart team would decide whether to do open surgery, intervention, or neither. We include Euroscore II to add more possibilities and realism to our cases, and to simulate physiological behaviours and complications based on some of these parameters.

### Environments

According to our clinical partners, the user should be able to navigate the visualisation of the heart model, toggle the visibility of heart regions, generate a new random patient, change PVA values and see the changes made to the model in real-time, read and change the Euroscore parameters, and save and load the generated model. Additionally, the user should be able to see the generated model inside the vCathLab using a fluoroscopy device, showcasing the generated patient. To implement these functionalities, we divided them into two environments. The first environment (authoring tool) includes everything related to generating the desired patient, and the second contains the vCathLab, which can be viewed on desktop and VR platforms.

**Fig. 3:**
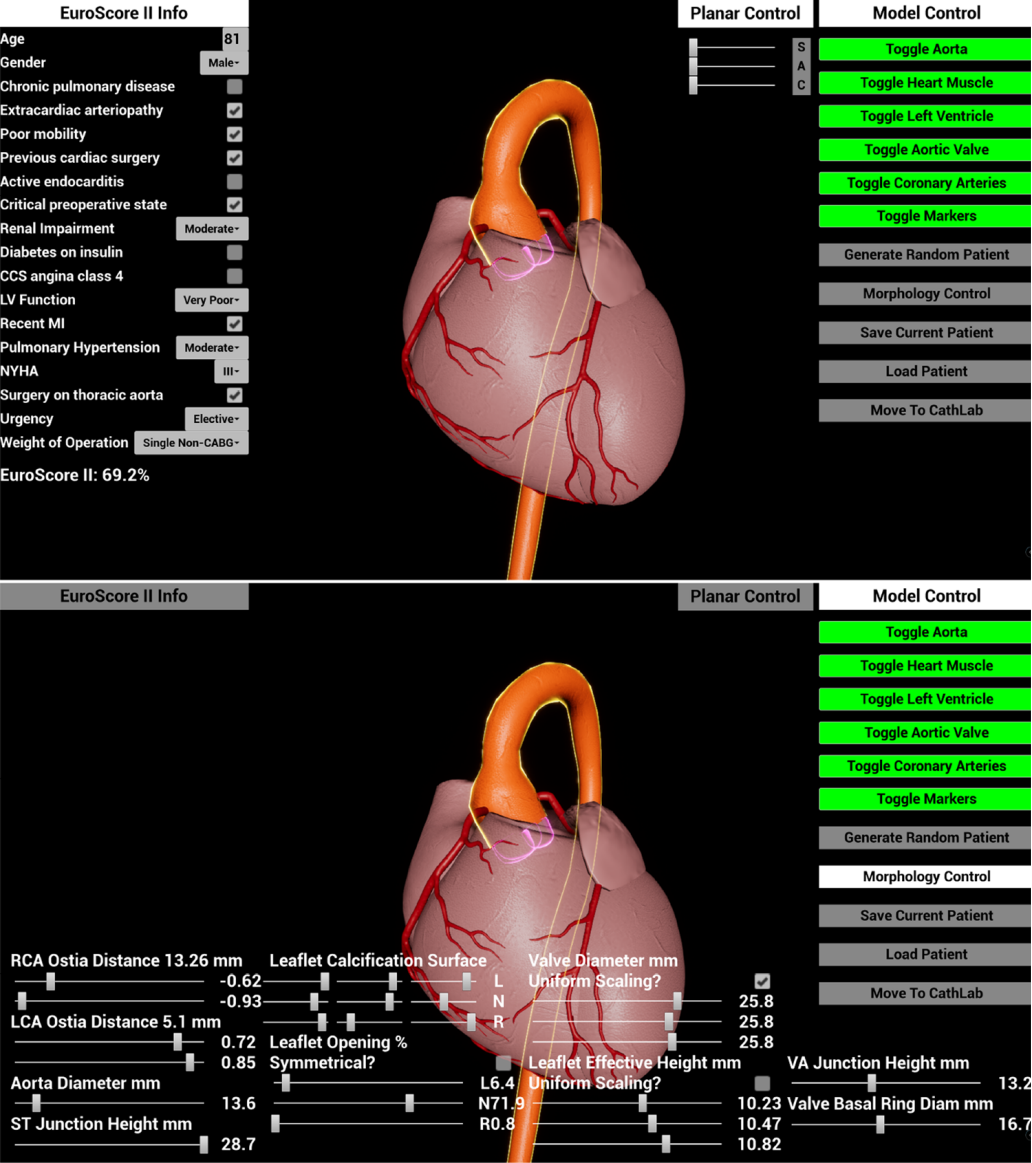
Authoring tool environment with top) Euroscore II information tab and bottom) Morphology sliders tab visible

#### Authoring tool

**Fig. 4:**
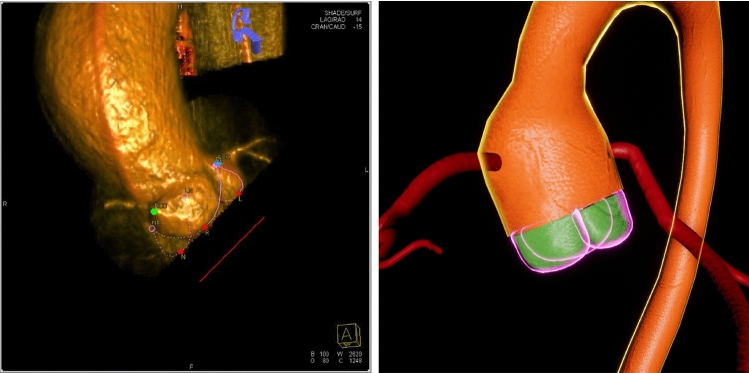
Medical design conventions as shown in left) Syngo Aortic Valve Guide (Siemens Healthcare GmbH, Germany) and right) the developed environment

This environment provides the means for generating the VPs, the main goal of this work, and consists of the heart model and the Euroscore and morphology parameters, which are accessed via four tabs (i.e. Euroscore II information, planar control, morphology control, and morphology sliders (see Fig. 3)). Our design choices, such as UI and visualisation designs, were inspired by conventions in medical literature and software (see Fig. 4). The model viewer can be navigated by rotation, panning, and zooming. To view the underlying structures of the heart, the visibility toggle feature was introduced, controlling each segment of the heart separately. Moreover, a planar control module aids the user by snapping the view to sagittal, axial, and coronal planes and providing clipping planes for each.

The Euroscore information can be viewed and modified through numerical inputs, dropdowns, and checkboxes, yielding new patient pre-condition criteria and changing the physiological behaviour. The Euroscore parameters are the counterpart to our morphology manipulation and therefore, a direct way of interacting with patient physiological simulation. This approach grants access to physiological modifications through limited, standardised, and known parameters, instead of an excessive amount of sliders and checkboxes. After changing each value, the Euroscore is updated.

One key feature of our system is the automatic generation of a new patient, both in anatomical and Euroscore parameters. These parameters are separated into two groups: dependent and independent (see Table 1). Accordingly, the pseudo-random algorithm generates the independent variables at first and, based on them, the dependent ones. For the Euroscore values, a probability multiplier was introduced that controls the outcome of each argument. These multipliers are provided by an expert and are approximated based on frequent cases. For some anatomical features, the parameters are controlled via multivariate regression functions that prevent unrealistic numbers if a specific condition is met, i.e. a narrower ST junction than the valve diameter. Each anatomical variable is picked from the ranges mentioned in Table 1).

Following the automatic model generation, the morphology control button enables the sliders tab, where the anatomical features can be modified. The tab provides the name, unit, and (in almost all cases) the amount of the anatomical parameter being affected. These sliders control groups of PVA values that are relative to one anatomical feature. In this way, we avoid redundancy and clutter in the workspace. We ensured that 2/3 of the screen is always dedicated to the display of the geometric model. Furthermore, the control tabs can be toggled to reduce workspace occupancy.

After creating the preferred patient, the user can move to the vCathLab to inspect the model in a fluoroscopy device. In parallel, a patient database is generated from the Euroscore values and communicated to PPE. After the PPE is initiated, the new environment loads and is ready for interaction. A save and load feature can help the user to revisit or utilise the model multiple times.

**Fig. 5:**
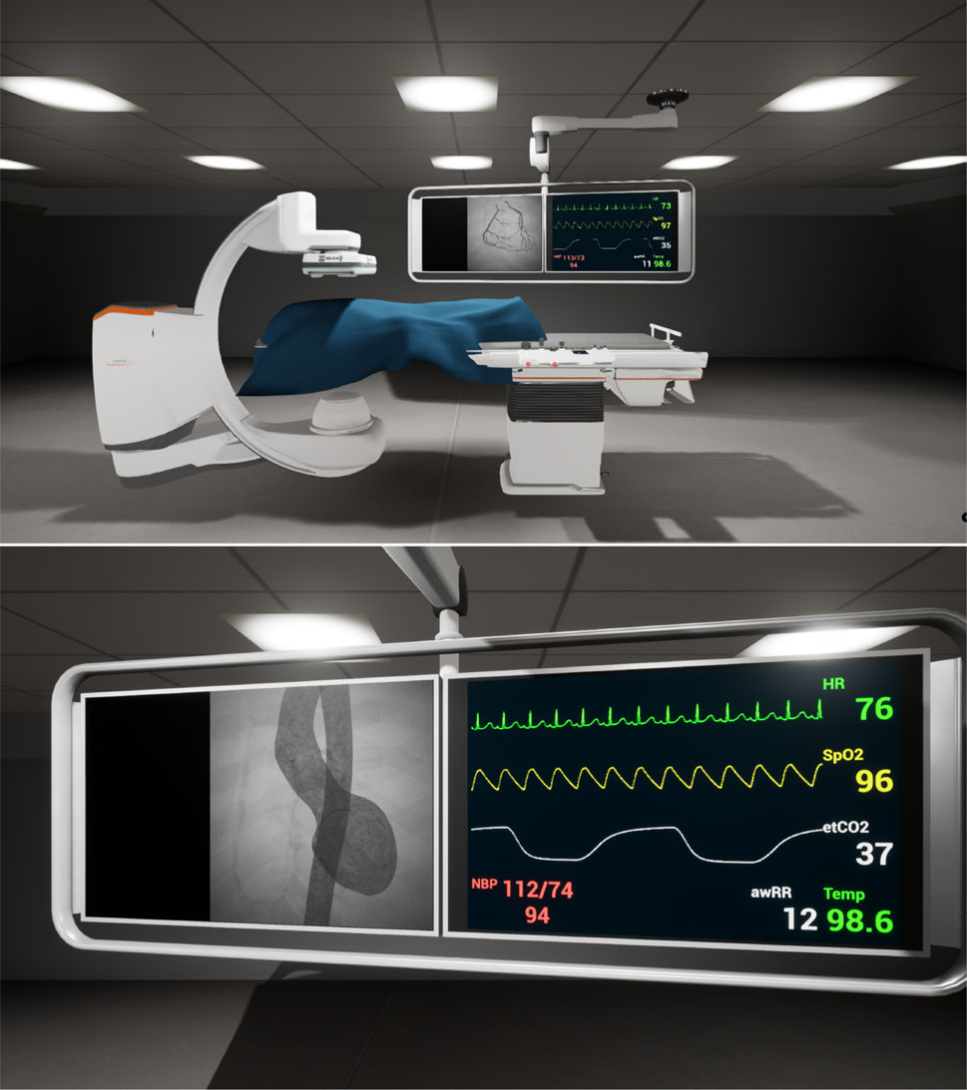
Virtual CathLab top) overview with contrasted coronary arteries, and bottom) monitoring screen and contrasted left ventricle and aorta

#### Virtual cathLab

While the focus of this work remains on the generation of a virtual patient using the authoring tool, we developed a VR environment to test the performance of the proposed method in a complex and computationally expensive scenario. This environment aims to provide the user with an immersive experience of a vCathLab. A virtual model of an ARTIS icono floor fluoroscopy system (Siemens Healthcare GmbH, Germany) is placed in this room with two monitors and a patient lying on the operation bed (see Fig. 5). The physiological parameters are viewed on the right monitor, and the live fluoroscopic images generated from the patient is rendered on the left monitor. The positions of the C-arm and the table can be manipulated by grabbing the virtual joysticks on the bedside control panel using headset controllers. The contrast agent injection can be triggered separately for coronary arteries, and for the ventricle and aorta. The virtual fluoroscopy device aims to represent a virtual twin of the system, helping the user understand the workflows and operation of such devices in different treatment scenarios. Hence, we recreated its interactions, movement, and imaging behaviours as realistically as feasible.

To generate the fluoroscopic images, a virtual camera with ray-tracing capabilities was attached to the virtual C-arm’s tube section. This camera captures only the translucent patient model in a dedicated lighting channel and applies a post-processing shader to achieve the fluoroscopic appearance. Finally, it renders the camera view to a render target texture, which is shown on the left monitor. Generating a replica of the imaging characteristics and of the pipeline of the fluoroscopy device was demanding and beyond the scope of this project. Therefore, we considered and implemented the relative graphical aspects in the post-processing shader to address the resource consumption of the virtual imaging system. These aspects are the grayscale visualisation, the grain noise texture and amount, the density of the tissue represented by alpha channel values, ray-tracing and scattering behaviour in volumes, the intensity of the X-ray source, the contrast agent movement and concentration, and the additive effects of digital subtraction.

### Evaluation

**Fig. 6:**
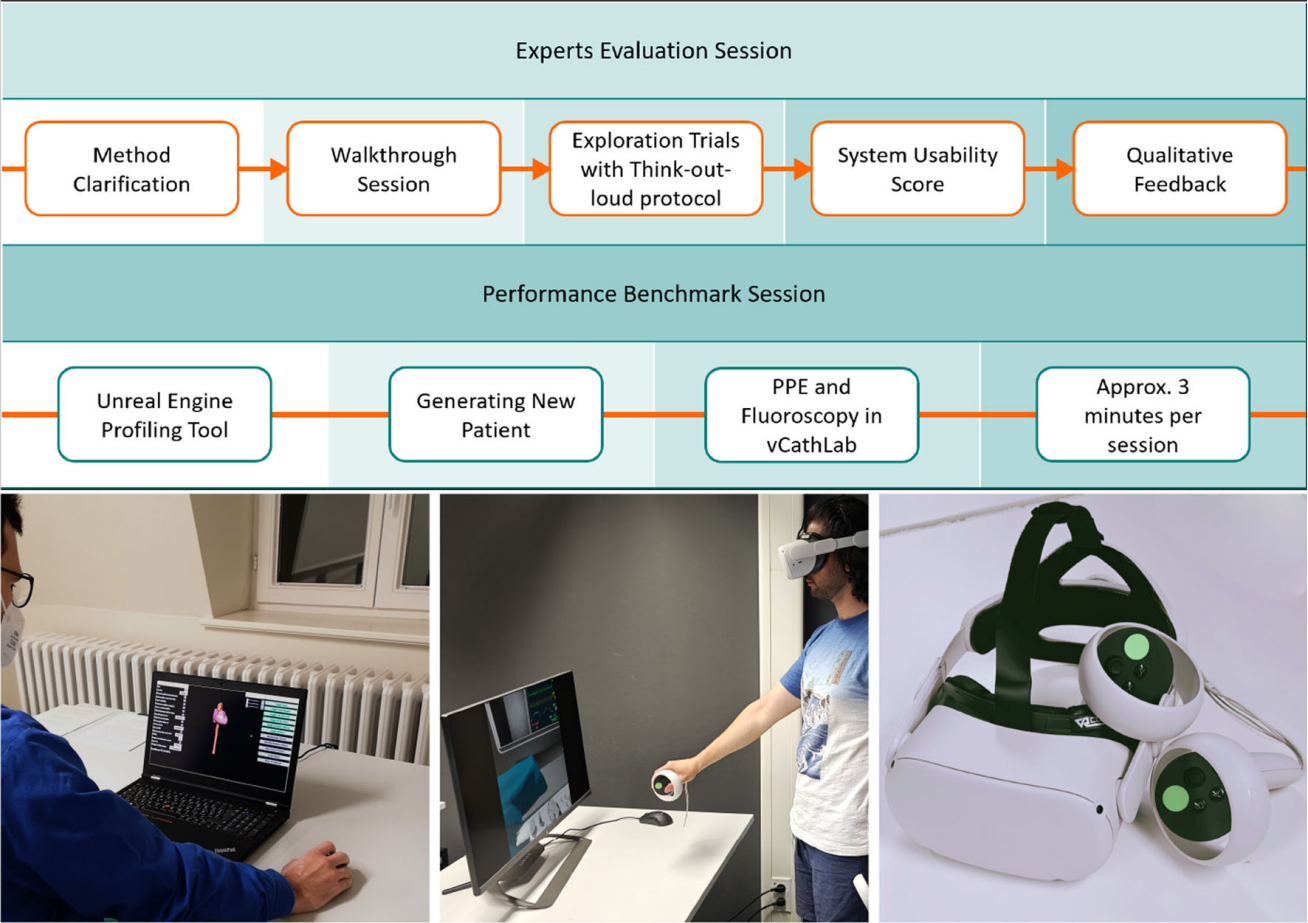
Top) Evaluation sessions overview Appendix A and 2.6, bottom left) an expert during the expert evaluation session, bottom centre) the user during a VR session, and bottom right) Meta Quest 2 device used for VR benchmarking

We evaluated our prototype through a subjective usability scoring, qualitative feedback from the experts, and performance benchmark in VR. Seven board-certified physicians (two cardiologists and five cardiac surgeons) took part in our evaluation. These physicians participated in the study under the project’s joint research agreement and were the primary representatives of our demography, i.e. trainers and trainees, with a professional interest and involvement in TAVR procedures. Five participants have been actively involved in teaching for an average of six years (from one to fifteen years); five perform an average of 43.6 TAVR procedures per annum (ranging from five to 100); and two of the cardiac surgeons were in training for TAVR procedures. The evaluation was carried out in two sessions formats, i.e. experts feedback and performance benchmark sessions (see Fig. 6).

We used System Usability Scale (SUS) Questionnaire [27] for our usability test. This well-established test is based on ten simple questions with Likert scale responses, enabling the user to subjectively rate their experience with the system from three aspects of effectiveness, efficiency, and satisfaction, on a scale of zero to 100 (worst to best). Bangor et al. devised an adjective rating scale for SUS to make the scale more readable and simpler to interpret [28]. This adjective rating scale consists of seven states, i.e. Worst Imaginable, Awful, Poor, OK, Good, Excellent, and Best Imaginable.

Frames-per-second (FPS), i.e. framerate, represents the smoothness of a virtual experience and thus the level of immersion in such environments. As the experience gets more complex and more computational power is required, e.g. in VR, the time needed to render one image from the virtual scene is increased, leading to lower FPS. The hardware capabilities, e.g. CPU and GPU, directly impact the FPS. However, the bottlenecks and resource consumption stay relatively the same across different hardware. Since FPS dictates input lag and perceived smoothness, it is a fundamental factor to the overall immersive experience and cybersickness state [29]. Furthermore, it encapsulates the relative computational processes, requirements, and bottlenecks in computer graphics. Hence, the FPS was selected as the measure for reporting the performance. FPS values can be recorded and analysed using benchmarking and profiling tools, which in our case is, provided by Unreal Engine.

To evaluate the performance of the generated patient in the VR environment, we ran a benchmark test using the Unreal Engine profiling tool. In each session, we generated a new random patient. We then transferred the model to our vCathLab, where we inspected it through the developed fluoroscopy and contrasting methods for approximately 3 min. During these tests, the PPE ran in parallel. The performance test was run by the VR developer to ensure all the functionalities and bottlenecks are assessed properly within the time frame.

## Results

Through SUS questionnaire, the application received an average score of 83.92 out of 100 (ranging from 72.5 to 95), and, accordingly, “Excellent” on the Bangor et al. adjective rating scale.

According to the participants’ feedback, this method could provide the proper context required for education and training of TAVR procedures while enabling the creation of various patients, including unique cases, quickly and straightforwardly. All participants found the proposed method valuable in the context of educational use cases. However, some improvements in the auto-generation algorithm and parameter ranges were suggested. Other recommendations relate to the interactions and GUI design which are independent of the proposed method.

**Table 2:** Performance results for benchmarked VR sessions

Sessions	Duration(s)	Total frames	Average FPS	+60 FPS up-time(%)
Session 1	181.9	14835	85.8	91.0
Session 2	181.6	14786	85.7	90.8
Session 3	181.1	15657	88.9	97.4
Session 4	183.8	15746	88.5	96.5
Average	182.1	15256	87.2	93.9

During the performance test, 61,024 frames were sampled across four sessions with a total duration of 729 s. An average of 87.29 (0.81–129.41) FPS was observed. We achieved an above 60 FPS count for an average of 93.97% of the time (90.82%–97.49%) (Table 2).

## Discussion

The prototype achieved positive results both in performance and usability. In terms of performance, an FPS below 50 is to be avoided at all costs [29]. Considering all the features running in parallel, we achieved promising results from our performance test. The extreme values were recorded mainly immediately after starting the runtime (minimum), immediately after loading the environment and before connecting to the SteamVR interface, and after ending the session (maximum). SteamVR and Unreal Engine development setting imposes an 89.99 FPS limitation. This can explain the average FPS being slightly below 90. Without these limitations, our system could achieve even higher FPS.

Regarding usability, subjective preferences of the participants in the UI features and design choices affected the SUS score negatively, i.e. a different control layout, improved visualisation, as well as including an orientation marker, a measuring system, and a colouring system. Experts suggested that future directions could include patient presets and backstories, haemodynamics, and imaging system angulations.

While Z-anatomy provides an acceptable basic model, the observed limitations of this model, in our case, are the exaggerated position of the right coronary ostia, undefined boundaries of the aortic annulus and VA junction, aortic sinus bulging, ST junction depiction, the diameter of the aorta, and the realistic details of the visualisations. We used Blender software to overcome these limitations, although, a more realistic 3D model of the heart can significantly improve the user experience by better visualisation of anatomical changes. Nevertheless, we verified the context-relevant anatomical correctness of our model with the help of our medical experts. We have done this during the development of the model and also during our evaluation phase on a bigger scale. The minimum acceptable correctness highly depends on the use case. Since we are establishing our method in educational context, the correctness requirements are more lenient compared to the clinical and treatment use cases.

Depending on the learning goals, anatomical modelling limitations can impact the learning and challenge achieving a high-fidelity training scenario. Physicians use visual cues, such as regional heart motions, contrast agent flow pattern, anatomical structures, as well as monitoring data to diagnose, assess, and control the patient’s state. Accordingly, understanding and recognising these patterns is a critical learning task for the trainee. However, some of these fine details can be lost during digital replications and simulations. For instance, creating a realistic representation of the fine differences resulted from heart wall thickness, valve size, aortic rigidness, haemodynamic flow, etc., is limited by the fidelity of the model, number of PVA groups, and the implemented animations. Minimising this loss is one of the challenges that conventional VP modelling tries to mostly address, while introducing other limitations, e.g. long development time and high costs.

The morphology control enables users to customise VPs with significant flexibility and variety; however, this modifiability can also lead to unrealistic cases [16]. The ability of PVAs to go beyond initially defined ranges further contributes to this risk. Though some control algorithms are implemented (see 2.3.1 and Table 1:Limited Dependency), a regression model was suggested to update and limit each parameter’s effective range. However, a reliable regression model containing all 18 parameters requires a large data set and is near unfeasible. Alternatively, Wang et al. [30] observed a correlation between many of these parameters and the patient’s body surface area (weight and height). Hence, creating a high-level regression model that controls each parameter based on an indirect correlation can be explored further.

While the current value ranges provide a general approach, they could be improved to represent a more realistic range for specific TAVR cases. The current modular design for changing these ranges enables this adaptation. In addition, implementing the extreme values while creating the PVA groups can act as a quality control, ensuring more extensive ranges and sensible deformations for the model. These case-specific ranges can also be gathered through literature surveys and medical measurements provided by the medical institutes, e.g. 3mensio (3mensio Medical Imaging BV, Netherlands) measurements for TAVR procedures.

Experts also suggested that adding several standard patient cases facilitates the recognition of the overall roles of defined parameters in such cases for trainees, and a basis for further model manipulation. These standard cases should have values based on actual patient datasets and be recreated through close collaboration with medical education experts for each procedure. Adding a list of saved patients can also contribute to these cases. In this way, the user can begin by selecting from available patients (a standard case or saved) and possibly with progressive difficulty before moving to randomised generation. Additionally, including a patient story can increase the engagement and immersion of the training scenarios. This story can be generated, in the future, based on the Euroscore parameters and through predefined decision trees or natural language processing methods [13]. Patient communication and interactions based on generative artificial intelligent models can also be a possible addition to this environment [31].

Haemodynamics of the heart and aorta are vital for the treatment in TAVR. Analysing haemodynamics pre-operatively can help physicians understand the patient’s condition and select the best treatment. These values can be generated and displayed using PPE, though further analysis is required to validate their accuracy. Moreover, a three- or four-dimensional visualisation of blood flow can significantly benefit our method. It may be possible to use deep learning methods for a fast approximation of the haemodynamic of the generated heart [32].

Another feature that was suggested was the including the visualisation of imaging system angulation. For TAVR procedures, the physician decides on this position while pre-operatively inspecting the 3D model in the control room. By displaying these values in the patient generation environment, we can add to the immersion and realism of the process while helping the user understand and practice utilising these values in the vCathLab. These values often differ in each case; hence, a proper solution for implementing this into the generated model could be further investigated.

We aimed to use a 3D model that reacts to a physiological simulation rather than it being handcrafted on a case-by-case basis. However, using a simulation-based approach also has its own drawbacks. Overall, the variety that exists in human’s anatomical features introduces immense complexity in all VP approaches, leaving its accuracy extremely dependent on its simulation method. While this challenge is the topic of many research, according to our experts and Massoth et al., visualisation of this variety within a relevant medical content (i.e. a procedure), even in lesser visual and simulation fidelities, can elevate the trainee’s understanding of the connection between each of the key medical features in the provided scenario [6, 33]. In addition, facing these situations helps reaching the risk management and decision-making learning goals for a significantly larger number of medical cases. Furthermore, using a simulation-based training enables the possibility of explorative learning, even with limited correctness. Free exploration can boost the understanding of different elements and their connections in a learning scenario, as well as keeping the users engaged with the environment reducing the number of repetitive tasks and the risk of boredom. These points all add value to our proposed method as a learning approach across various medical educational context. Although this work focuses on TAVR procedures, the proposed approach can also be used for other organs and scenarios to demonstrate the importance of anatomical feature variations in training applications.

During the development of this method, some primary didactic design factors such as unsupervised and supervised patient generation were taken into account. Accordingly, the virtual patient case can be designed by the trainee or an expert trainer. Therefore, this approach can be crucial in educating medical trainees through adaptive learning methods. Hence, a treatment difficulty in the patient generation algorithm can benefit the method. Accordingly, users could start their training from a beginner’s level and gradually build up their skills. This should be followed by a well-designed evaluation system that would report the user’s progress in a detailed manner with appropriate feedback systems during and after each session and long-term progress tracking [34, 35]. Overall, further investigation is required to fully exploit the didactic potentials of the proposed authoring tool and developing a tutoring system.

## Conclusion

We designed and developed an authoring tool and a vCathLab Environment for creating and examining a wide variety of virtual patients for TAVR procedures in a streamlined and resource-friendly manner, compared to the existing methods. This approach uses Euroscore, PPE, and PVA to generate new physiological and anatomical behaviour. Evaluation of our method yielded acclaiming results, mainly on its modifiability and variability. In addition, the ease-of-use was another positive remark. The compelling performance test results express the suitability of this solution for VR-based education and training. The addition of a well-designed evaluation system, more case-specific customisation capabilities, and improved patient data and communication scenarios were also identified as potential future directions.

## Supplementary Information

Below is the link to the electronic supplementary material.

Supplementary file 1 (mp4 38738 KB)

## Data Availability

The methods and information presented in this work are based on research and are not commercially available.

## Appendix A: Expert evaluation sessions description

Before interacting with the environment, the goals of the prototype are clarified to each user. Afterwards, they are asked to perform simple tasks regarding the available features while receiving verbal instruction and being guided through it, i.e. model navigation, planar control, interaction with the Euroscore II calculator, model control tab, random patient generation, and morphology control tab, sequentially. In the next step, they are asked to create two more random patients and tailor them to their preferences, using the Euroscore and morphology control features, as they describe their experience using the thinking-aloud protocol. Finally, they are asked to rate the system’s usability and provide qualitative feedback on its features.
